# Effects of protective step training on proactive and reactive motor adaptations in Parkinson’s disease patients

**DOI:** 10.3389/fneur.2023.1211441

**Published:** 2023-10-24

**Authors:** Thurmon Lockhart, Chris Frames, Markey Olson, Seong H. Moon, Dan Peterson, Abraham Lieberman

**Affiliations:** ^1^Locomotion Research Laboratory, School of Biological and Health Systems Engineering, Arizona State University, Tempe, AZ, United States; ^2^Muhammad Ali Movement Disorders Clinic, Barrow Neurological Institute, St. Joseph’s Hospital and Medical Center, Phoenix, AZ, United States; ^3^Gait and Balance Dysfunction Laboratory, College of Health Solutions, Arizona State University, Tempe, AZ, United States; ^4^Department of Veteran’s Affairs, Phoenix, AZ, United States

**Keywords:** Parkinson’s disease, accidental falls, protective step training, motor learning, gait and balance, motor adaptability, feedforward and feedback, physical therapy

## Abstract

The aim of this study was to investigate to what extent PD affects the ability to walk, respond to balance perturbations in a single training session, and produce acute short-term effects to improve compensatory reactions and control of unperturbed walking stability. Understanding the mechanism of compensation and neuroplasticity to unexpected step perturbation training during walking and static stance can inform treatment of PD by helping to design effective training regimens that remediate fall risk. Current rehabilitation therapies are inadequate at reducing falls in people with Parkinson’s disease (PD). While pharmacologic and surgical treatments have proved largely ineffective in treating postural instability and gait dysfunction in people with PD, studies have demonstrated that therapy specifically focusing on posture, gait, and balance may significantly improve these factors and reduce falls. The primary goal of this study was to assess the effectiveness of a novel and promising intervention therapy (protective step training – i.e., PST) to improve balance and reduce falls in people with PD. A secondary goal was to understand the effects of PST on proactive and reactive feedback responses during stance and gait tasks. Multiple-baseline, repeated measures analyses were performed on the multitude of proactive and reactive performance measures to assess the effects of PST on gait and postural stability parameters. In general, the results indicate that participants with PD were able to use experiences with perturbation training to integrate and adapt feedforward and feedback behaviors to reduce falls. The ability of the participants with PD to adapt to changes in task demands suggests that individuals with PD could benefit from the protective step training to facilitate balance control during rehabilitation.

## 1. Introduction

Parkinson’s disease (PD) is a neurological disorder characterized by bradykinesia, tremor, rigidity, postural instability and, affects an estimated 1 million individuals in the US. Postural Instability and Gait Dysfunction (PIGD), a subset of PD symptoms describing impaired standing posture and balance, bradykinetic gait features, freezing of gait (FOG), and falls, is a disabling condition that, unlike other cardinal features of PD, is often not adequately treated by dopaminergic medications. Fall incidence rates among the PD population are estimated to range as high as 50–70%, with many individuals suffering recurrent falls, and these falls are a major cause of injury and disability. It is estimated that healthcare expenditures related to these falls exceeded $27 billion in 2013 (1). As the population of older adults (>65 years old) in the US increases over the coming decades, reaching a projected 98 million by 2060 (2), the rates of PD and associated falls are expected to rise dramatically. Although modern medicine and new medical technologies offer enormous potential to improve the diagnosis and treatment of many symptoms, falls still represent a major and largely untreated problem for PD patients. While pharmacologic and surgical treatments have proved largely ineffective in treating PIGD thus far (3–9), studies have demonstrated that therapy specifically focusing on posture, gait, and balance may significantly improve these factors and reduce falls (10–13).

Perturbation-based balance training, or protective step training (PST), defined as balance training using repeated, external perturbations, is one such method of therapy that has demonstrated improvements in balance and fall recovery in multiple populations (14). Several studies have observed decreased fall rate and an increased ability to recover from a fall upon repeated exposure to a perturbation in healthy controls (15, 16). Investigators have reported that adaptations to avoid falling can be modulated *via* both feedforward (predictive) and feedback (reactive) mechanisms (17). Predictive mechanisms of recovery involve changes to gait parameters such as base of support, trunk angle, and velocity, that may reduce the magnitude of the required balance recovery response upon delivery of a perturbation. Reactive mechanisms of improvement may involve earlier detection of perturbation, likely to require recovery response, and improved motor responses triggering increased relevant muscle action and fewer maladaptive movements following perturbation (17). Studies suggest that reactive balance may not be entirely intact in PD, but that learning is still possible (3–9), making it important to study the effects of PST in this population.

Recent literature for PST in populations with PD is represented in Table 1. Studies indicate that PST may be useful in improving gait and postural control that precipitate future falls (23), however, there is a lack of consistency regarding the specific improvements and whether those improvements can effectively transfer from a rehabilitation setting to activities of daily living. Studies have shown that training is more effective if it is specific to the skill to be improved (23), and while perturbation-training in PD is ongoing, the majority of research is not task-specific and only a few studies have attempted to replicate common causes of falls (e.g., slips and trips) (19–21, 24). To this end, the specificity of PST is in need of further study to determine what types of perturbation are most effective at inducing adaptive response and what intensity, frequency, and duration of perturbation training sessions are required for these results to be retained.

**Table 1:** Characteristics of Gait Perturbation Studies in PD.

Publication	Sample size	Perturbation Training	Protocol	Perturbation Type	Outcomes
Oates et al. (18)	*n* = 8 PD *n* = 10 age-matched controls	Overground walk with slip perturbation	Single session 15 walk trials 1 unexpected slippery stop 5 planned stops 5 cued stops	Unexpected slip perturbation during GT^1^	PD showed slower, wider steps and less stability Feedforward adaptations: shorter, wider step, modified MOS^2^. Feedback adaptations: longer, wider step.
Steib et al. (19)	*N* = 38 PD	Treadmill walking	3 months 16 sessions total 8 weeks of treadmill walking for 30 min	Three-dimensional tilting movements to the treadmill	No effect with perturbation training on gait and balance in PD patients.
Klamroth et al. (20)	*N* = 39 PD	Treadmill walking with tilting	1 session, 20 min of treadmill walking and 10 min	Three-dimensional tilting movements to the treadmill floor	Increased walking speed (overground) in PBT^3^ group compared to control group. Gait variability during treadmill walking significantly decreased after walking with PBT
Martelli et al. (21)	*N* = 18 subjects PD subjects (*n* = 9) HOA^4^ (*n* = 9)	Treadmill walking with cable perturbations	Single session, 30 minutes Treadmill walking: 9 blocks of 8 AP or ML perturbations by cables	AP & ML push or pull perturbations	Reduced stability in AP direction and proactive adaptations. Reported short-term after-effects of increased gait stability.
Hulzinga et al. (22)	*N* = 52 PD subjects FOG^5^ (*n* = 22) Non-FOG (*n* = 30)	Treadmill training: SBT^6^ & TBT^7^	*Training:* *For 4 weeks, 3x per week.* *Three 1-min walking trials: (1) TBT (baseline); (2) SBT (to assess early and late adaptation); and (3) TBT (to assess early and late de-adaptation)* Tests: 1 week prior (pre), 1 week after (post) and 4-weeks (post).	*Asymmetrical gait-speed perturbations: SBT had 50% reduction in speed on one side.*	SBT-training improved gait adaptation more than TBT, effects that were sustained at follow-up and during dual tasking. Gait speed and step length improved with SBT & TBT. Gait adaptation did not transfer to over-ground turning speed.

Gait termination^1^; margin of stability^2^; perturbation training^3^; healthy older adults^4^; freezing of gait^5^; split-belt treadmill training^6^; tied-belt treadmill training^7^.

The primary goal of this study was to assess the effectiveness of two kinds of PST (anterior translations of a split-belt forceplate during (1) forward gait and (2) static postural stability tasks) to improve balance and stability in people with PD by better understanding the effects of PST on proactive and reactive feedback responses during stance and gait. We hypothesized that PD patients will be able to learn in an explicit, feedforward manner, adjusting base of support prior throughout walking trials to prepare for unexpected perturbation, but may be unable to improve reactive response variables such as reaction time and strategy. This work may enhance the clinician’s ability to treat balance/gait disturbances leading to falls in people with PD utilizing protective step training.

## 2. Materials and methods

### 2.1. Participants

Twelve participants diagnosed with PD by a movement disorders neurologist were recruited for this study (age = 62 ± 7.1; 9 males, 3 females). Participants were included in the study if they were able to ambulate without assistance, had no known neurologic, cardiovascular, or orthopedic deficit that could significantly impact cognition and functional performance (Mini-Mental Status Examination <25), and had a Hoehn & Yahr (H&Y) score between II-III. Subjects were excluded if they exhibited functionally disabling dyskinesia or dystonia, orthostatic hypotension, neurosurgical intervention (deep brain stimulation), and any significant musculoskeletal or metabolic disorders. All subjects were examined during the “on” dopaminergic medication state, having taken their last dose approximately 1–1.5 h prior to testing. Disease severity and clinical scales of symptoms were tested in the “on” state utilizing the H&Y scale (25) and the motor subscale of the International Parkinson and Movement Disorder Society-Sponsored revision of the Unified Parkinson’s Disease Rating Scale (MDS-UPDRS Part III). Subjects in the present study had an average H&Y score of 2.7 and an average disease duration of approximately 3.5 ± 3.1 years. Prior to testing, subjects were randomly assigned to two groups. One group started with postural perturbation (PP) training (during stance), while the other group began with gait perturbation (GP) training before crossing over (during walking). During this onboarding period, self-reported and observed leg dominance in bilateral mobilizing was utilized to determine the dominant leg that will be perturbed in the walking trials. Investigators described a scenario for the participant in which they were asked which leg they would use to kick a ball over the ground. This study (experimental procedures and design) was approved by the Institutional Review Board at Arizona State University and performed according to the declaration of Helsinki. All participants provided written informed consent prior to data collection.

### 2.2. Study design

**Figure 1:**
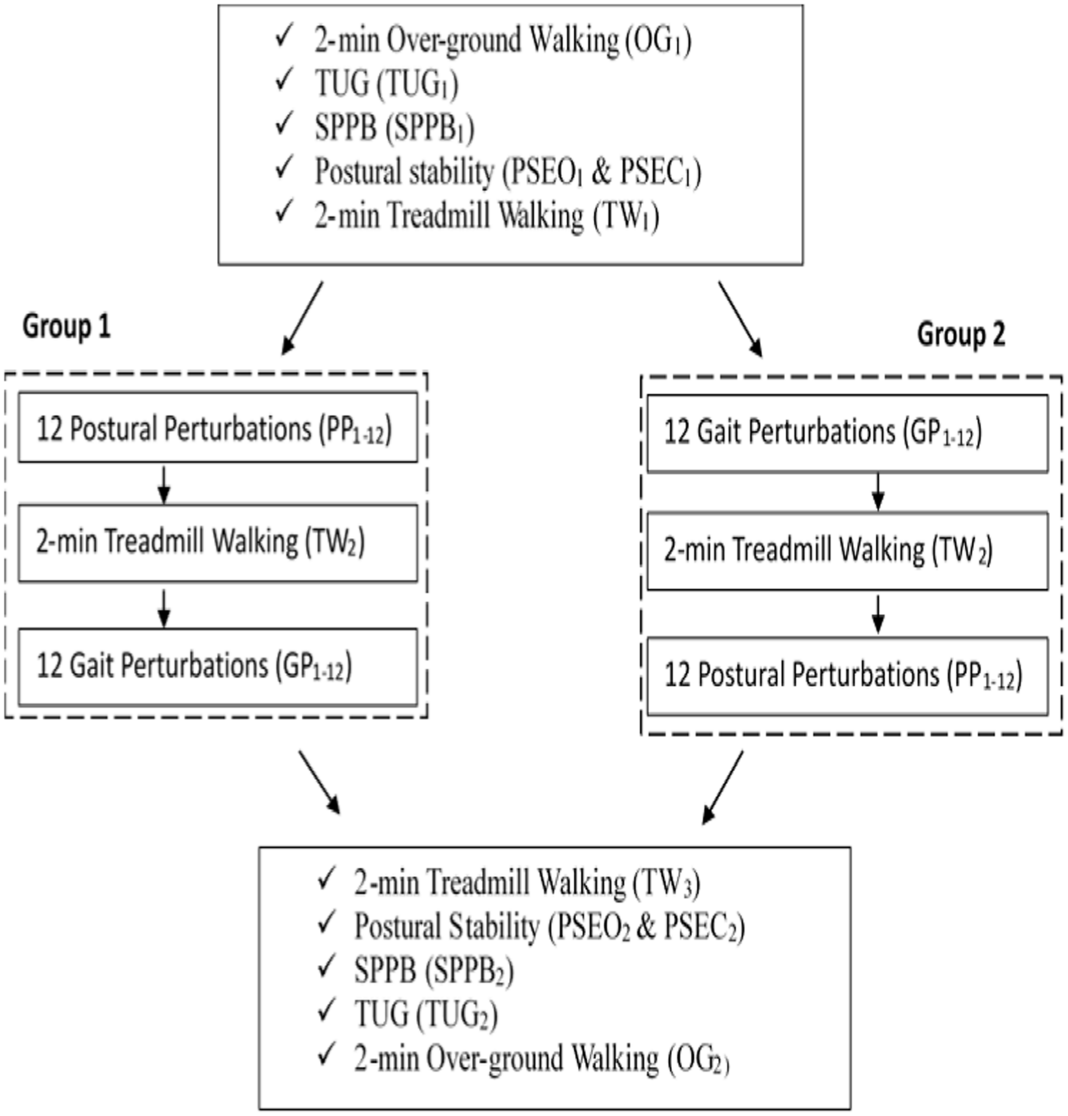
Schematic of study design.

**Figure 2:**
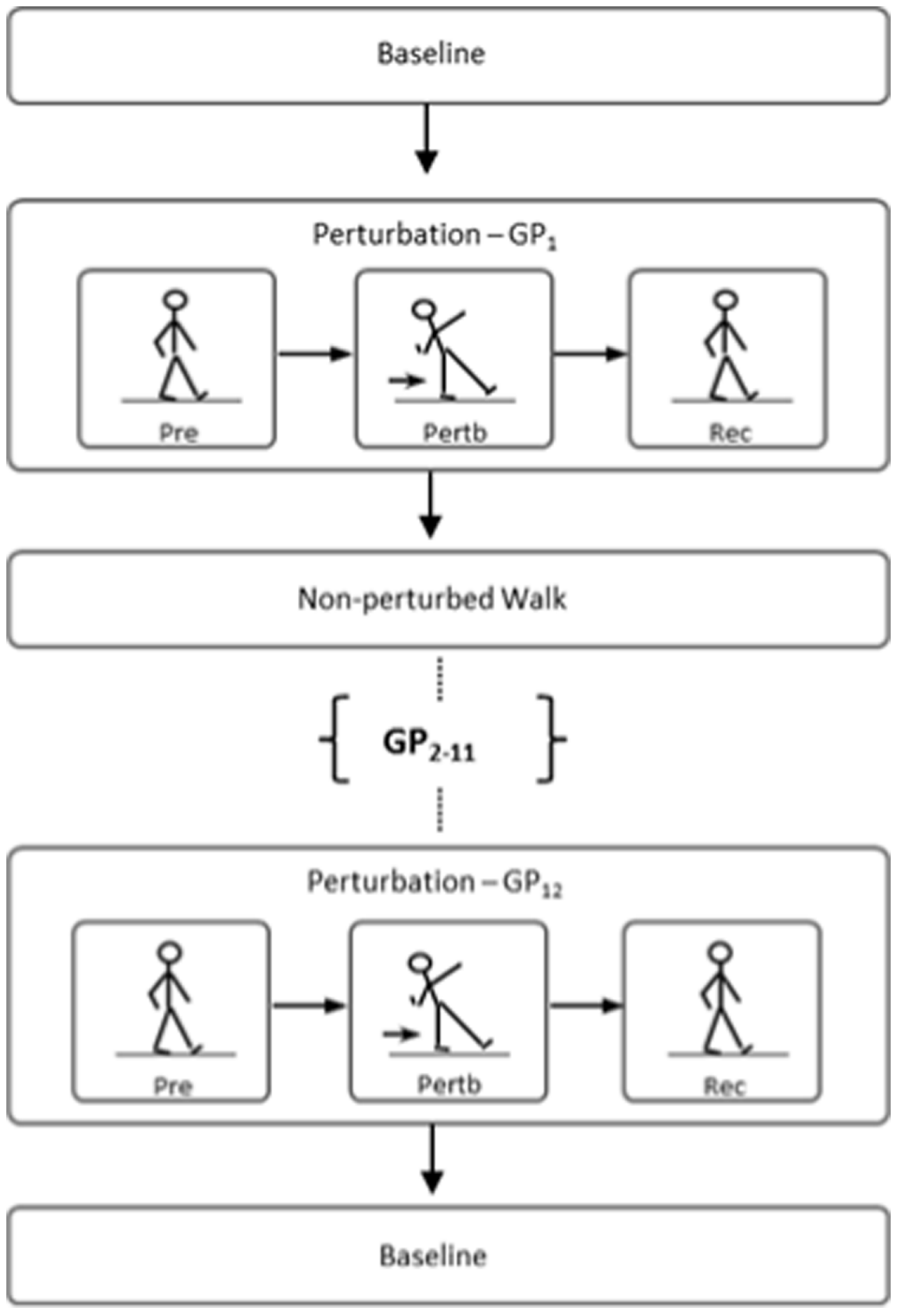
Illustration of comparison made between novel recovery from the first perturbation (i.e., GP_1_ or PP_1_) vs. trained recovery from the last two perturbations (i.e., GP_11,12_ or PP_11,12_).

Prior to baseline testing, two 3-min walking sessions were given to the participants to familiarize themselves with the treadmill and the laboratory environment. The first session was primarily for familiarizing and adapting to the treadmill. Following this session, the participant was given a rest period and encouraged to ask questions or bring up any concerns with the task. The second session was used to standardize the participant’s preferred walking speed (PWS) that they would be using throughout the trials. In this session, walking speed was increased incrementally until participants indicated that the speed was consistent with their normal walking speed. The walking speed was then increased in 0.1 m/s increments until the participant expressed discomfort or reported the speed to be inconsistent with their normal walking speed. Following the familiarization period, the protocol began with a series of baseline tests: 2-min of overground walking (OG_1_), Timed Up-and-Go Test (TUG_1_), Short Physical Performance Battery Test (SPPB_1_), postural stability in both the eyes-open and eyes-closed conditions (PSEO_1_, PSEC_1_), and 2-min of treadmill walking (TW_1_). Following baseline trials, participants in the first group commenced postural perturbation (PP) training during stance while the second group commenced gait perturbation (GP) training during walking. Both groups were instructed to maintain their balance and avoid a fall when introduced to unexpected perturbations. Upon completion of the respective training paradigms, a ‘washout’ period was introduced in which the groups performed a second 2-min treadmill walking trial (TW_2)_ to observe any after-effects from baseline. Following this period, the two groups crossed over and commenced the alternate perturbation training paradigms. Finally, both groups performed a final 2-min treadmill walking trial (TW_3_) along with the post-training tests performed during baseline testing: OG_2_, TUG_2_, SPPB_2_, PSEO_2,_ and PSEC_2_. All perturbations occurred on each participant’s dominant leg. A schematic of the study design is presented in Figure 1. Figure 2 illustrates comparisons made in this study.

### 2.3. A protective step training during stance and gait

Perturbation training protocol was based on previous study on young and older adults without PD (26–29). The protective step training protocol during walking, i.e., gait perturbation (GP) training, consisted of continuously walking on a treadmill with 12 blocks (GP_1-12_) of unexpected perturbations. Participants began walking unperturbed for a baseline period of approximately 15 s. The subsequent 10 s following the baseline period are designated as the perturbation window: a 10-s window in which the subject is given an unexpected anterior translation of the right treadmill belt (acceleration of 10 m/s^2^; duration of 0.2 s) at the instant of right heel contact, i.e., the perturbed step. This translation resulted in the displacement of the subject’s COM, in which participants were instructed to restore their balance. Following the perturbed step and the subsequent recovery duration, participants walked unperturbed until they were able to match their preferred walking speed again.

The protective step training protocol during stance, i.e., postural perturbation (PP) training, evaluated standing balance maintenance given 12 unexpected perturbation blocks (PP_1-12_). Participants were instructed to stand upright with their arms by their sides and look straight ahead. The training began with participants in quiet stance for approximately 15 s. Following this static period, a simultaneous anterior translation of both treadmill belts was induced with an acceleration of 8 m/s^2^ and duration of 0.1 s. Akin to the GP training, the resulting platform translation resulted in the displacement of the subject’s COM, in which participants were instructed to restore their balance.

### 2.4. Experimental setup

**Figure 3:**
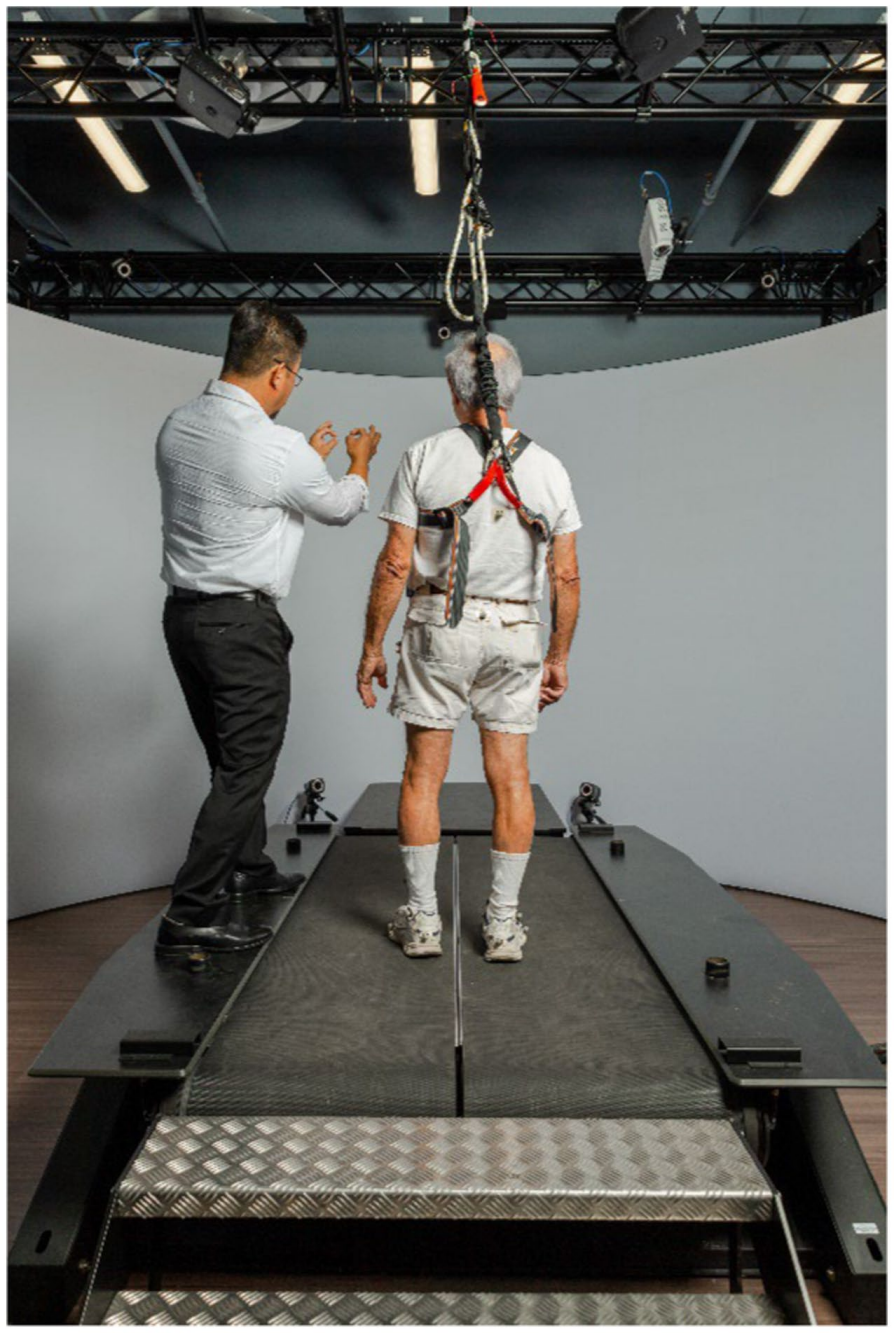
GRAIL system with dualbelt instrumented treadmill.

GRAIL system (Gait Real-time Analysis Interactive Lab, Motek Medical, Amsterdam, the Netherlands) was utilized to simulate both types of (i.e., during standing and walking) perturbations. GRAIL consisted of a dual-belt instrumented treadmill equipped with dual embedded force plates in a speed-matched virtual environment projected on a semi-cylindrical screen (during the experiment, the virtual environment was turned off and a blank wall was projected to avoid any visual perturbation effects) (Figure 3). Subjects were equipped with standardized footwear to minimize experimental confounds, as well as a full-body harness tethered to an instrumented safety system that supported their full weight. The dual force plates embedded in the treadmill belts were utilized to collect data during postural stability trials. Lower body kinematics was recorded using 12 Vicon cameras (100 Hz; Vicon Bonita, Vicon, United States) with a modified Helen-Hayes marker set, including 25 reflective markers, which were placed in accordance with the lower body Vicon full-body Plug-in-Gait model. Motion capture data was filtered using a fourth-order low-pass Butterworth filter and a cut-off frequency of 6 Hz. Accordingly, force plate data was filtered using a fourth-order-low-pass Butterworth filter with a cut-off frequency of 10 Hz, to eliminate extraneous measurement noise. Nonlinear measures – applied during TW_1_, TW_2_, and TW_3_ – were implemented to estimate the structure of variability, e.g., the scaling behavior (scaling exponent α) of stride intervals and the signal regularity (MSE) of center of mass (COM) velocities – were unfiltered during the analysis. All analysis was performed using custom Matlab routines (The Mathworks, Version 2016a).

### 2.5. Data analyses

Feedforward responses from gait were analyzed from the 15 s of unperturbed walking prior to each perturbation. Spatiotemporal parameters and gait variability were extracted from the 10 steps prior to the perturbation, while dynamic stability was examined at the final heel contact before the perturbation (pert_hc_). Adaptive behavior was determined by comparing responses from GP_11_ and GP_12_, with GP_1_. Regarding PP training, predictive postural adjustments were assessed from the 15 s of quiet standing prior to the perturbation. The whole-body center-of-mass (COM) dynamics, base of support (BOS), and angular kinematics of the trunk, knee, and ankle in the sagittal plane delineated any changes to feedforward control prior to a perturbation. Adaptive behavior was assessed by comparing responses from the last two perturbation blocks (PP_11_ and PP_12_)-Post-test-with the initial block (PP_1_) – Pre-test. Joint angles were measured from pert_hc_, while joint angle range of motion (ROM) was standardized from the minimum and maximum angles within the dominant leg’s normalized gait cycle just prior to pert_hc_. Angles were calculated by the segmental method for determining 2D joint angles by the means of cardan sequences and a 6 degrees of freedom model. Relative angle was determined between the local coordinate systems of each proximal and distal segment. The angles chosen for sagittal plane analysis were trunk flexion/extension (measured as the angle between a vertical line, perpendicular to the ground, bisecting the sacrum and a line bisecting the thoracic spine), knee flexion/extension (defined by the long axis of the tibia with respect to the long axis of the femur), and ankle plantar and dorsiflexion determined by the shank and foot segments. For knee joint angles, full extension was defined as zero degrees and movement into flexion being positive. Regarding ankle angles, zero was set at 90° to delineate plantarflexion and dorsiflexion. Plantarflexion was set as the negative degrees. In the frontal plane, lateral trunk flexion was measured as the angle between a vertical line bisecting the contralateral ASIS (perpendicular to the ground) and a line from the ASIS to the AC joint marker. Table 2 provides further operational definitions of the feedforward parameters.

**Table 2:** Definition of parameters.

Parameters	Definition
Feedback parameters	
Recovery period (Time_Rec_)	Time elapsed from perturbation onset until the zero-cross of the AP COM velocity (recovery point).
Latency (Time_Latency_)	Time elapsed between perturbation onset and the initial reactive response from the AP force plate [ms].
Recovery step time (Time_1stStep_)	Time elapsed between perturbation onset and the first recovery step of the contralateral foot [ms] – Step calculated by the zero-cross of AP heel marker velocity.
Path length (PL)	The total length of the COP trajectory in the AP & ML directions [mm].
Normalized path length (nPL)	Path length normalized by its variance. Measures the coordinative structure of the COP (AP & ML); reflects the number of times there is a change in direction.
Velocity	Velocity of COP calculated by Path Length over Time_Rec_ [mm/s].
Margin of stability (MOS_ML_)	Distance between the lateral boundary of the BOS at heel contact (heel marker of the leading foot) and the extrapolated COM.
Margin of stability (MOS_AP_)	Distance between the anterior boundary of the BOS at heel contact (toe marker of the leading foot) and the extrapolated COM.
Root mean square (RMS)	Statistical measure of COP magnitude in the AP & ML directions.
Feedforward parameters	
Trunk angle	Trunk flexion/extension from the sagittal plane (positive = flexion; negative = extension) [deg].
Knee flexion angle	Knee flexion angle from the sagittal plane (positive = flexion; negative = extension) [deg] from the dominant leg.
Ankle angle	Dorsiflexion/plantarflexion of the ankle joint in the sagittal plane (positive = plantarflexion; negative = dorsiflexion) [deg] from the dominant leg.
Base of support (BOS_ML_)	Horizontal stride width during the double-support phase of gait. Stance width during standing perturbations.
Base of support (BOS_AP_)	Step length during the double-support phase of gait. Stance width during static standing.
Margin of stability (MOS_ML_)	Distance between the lateral boundary of the BOS at heel contact (heel marker of the leading foot) and the extrapolated COM.
Margin of stability (MOS_AP_)	Distance between the anterior boundary of the BOS at heel contact (toe marker of the leading foot) and the extrapolated COM.
Heel contact velocity (HCV)	Instantaneous AP heel velocity calculated utilizing AP heel velocities at the foot displacement 1/100 s (Δt) before and after pert_hc_ [mm/s] (Lockhart et al., 2003) from the dominant leg. vpert_hc_ = [*x*(*i* + 1) – *x*(*i*–1)]/2Δt
Gait parameters	
Gait cycle time	Time elapsed between two consecutive heel contacts of ipsilateral foot.
Step time	Time elapsed from the heel contact of one foot to heel contact of the subsequent contralateral foot.
Stance time (RST & LST)	Time elapsed from the heel contact to the toe-off of a single footfall [s]. Calculate left (LST) & right (RST).
Double support time (DST)	Time elapsed from the heel contact of one foot to the toe-off of the contralateral foot. The sum of two periods of double support in the gait cycle [s].
Root mean square (RMS)	Statistical measure of the COM magnitude in the AP, ML, or V direction compared to the total trunk acceleration magnitude.
Coefficient of variation (CV)	Measure of variability normalized to the mean of a specific parameter [%]. CV = (SD/Mean) × 100

**Figure 4:**
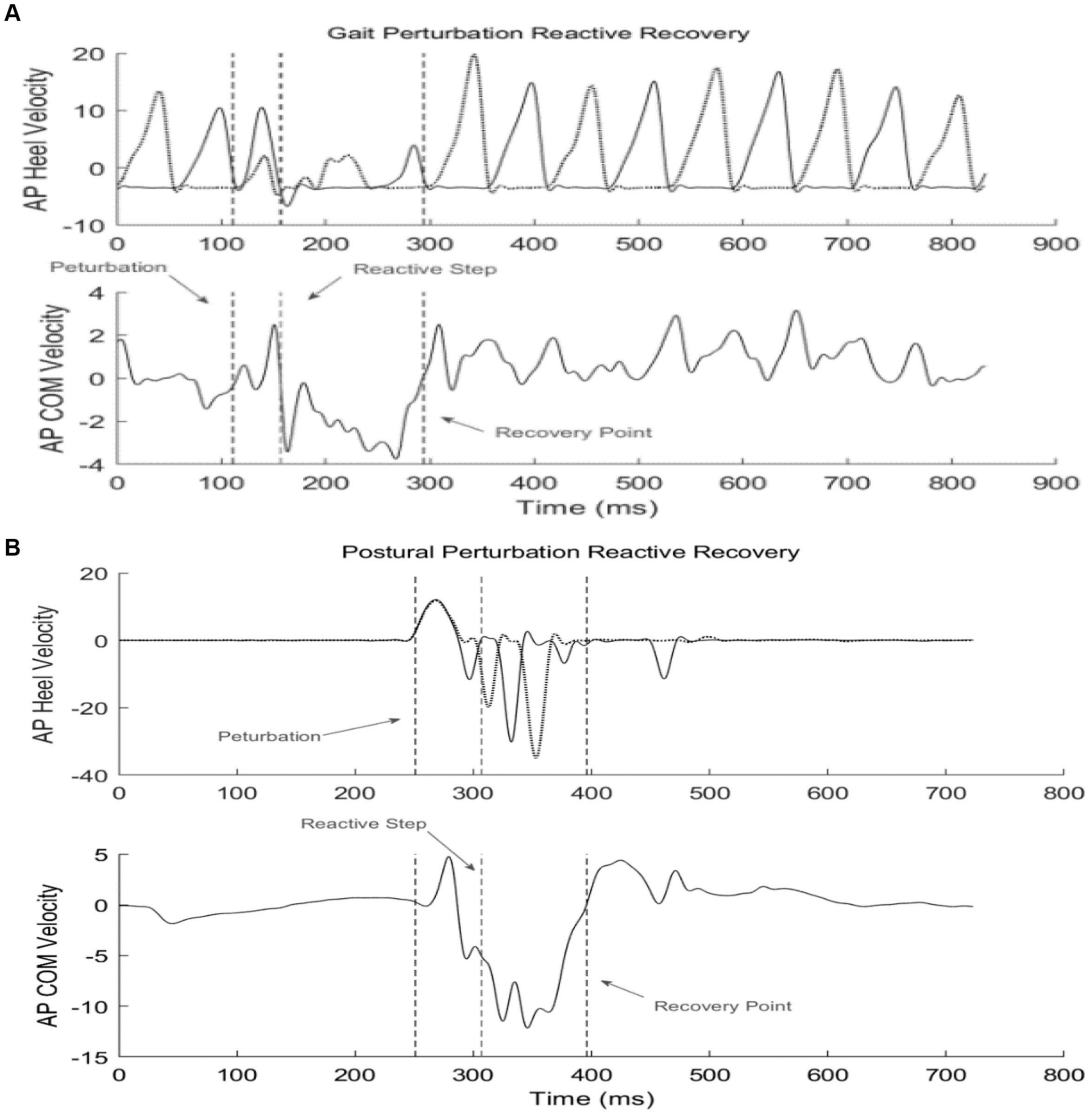
Example of reactive recovery dynamics given a perturbation during stance or gait. The 1st dotted line identifies perturbation onset; the 2nd line identifies the reactive recovery step (rechc); the 3rd line marks the termination of the recovery period. **(A)** Gait perturbation example; **(B)** Postural perturbation example.

Reactive feedback responses from GP training were examined by identifying the contralateral recovery step (rec_hc_) immediately following the perturbation (Figure 4A). Dynamic stability of the first recovery step from perturbation blocks (GP_11_ and GP_12_) was compared with the dynamic stability of the initial block (GP_1_), to evaluate adaptive feedback control. For PP training, reactive modifications from the first recovery step after the perturbation (rec_hc_) was identified as the initial recovery mechanism. The end of the recovery period was identified as the zero-cross point of the COM velocity in the anteroposterior direction (Figure 4B). Table 2 outlines the feedback parameters used to evaluate subject performance.

Dynamic stability was calculated by the margin of stability (MOS), which measures the movement of the COM relative to the base of support (30). Specifically, MOS in the anteroposterior (AP) direction, was determined by the distance between the anterior boundary of the BOS at heel contact (toe marker of the leading foot) and the extrapolated COM (30); MOS in the mediolateral (ML) direction was calculated as the difference between the lateral boundary of the BOS at heel contact (heel marker of the leading foot) and the extrapolated COM. An increased MOS indicates the COM is further within the BOS, while decreased MOS indicates COM is nearer to the limits of the BOS.

Combined after-effects of the perturbation training were evaluated from continuous gait on the treadmill, both before and after testing (TW_1_ and TW_3_). Measures of gait variability, complexity, and smoothness were employed to determine the sensitivity of the pre-and post-training effects. Variability was assessed by the RMS of COM accelerations (AP, ML, and V) along with statistical measures of variability from spatiotemporal gait parameters: Standard deviation (SD) and coefficient of variation (CV). CV denotes the variability of a specific gait parameter normalized to its mean value; it is represented as a percentage (CV=SD/mean × 100). Gait complexity was measured by multiscale entropy (MSE). MSE is a regularity measure developed by Costa et al. (31) that quantifies the information content of the gait signal (COM velocities in the AP, ML, and V directions) over a range of physiologically relevant time scales while sample entropy is computed for every consecutive coarse-grained time series. The entropy values are then plotted as a function of the time scales in which the area under the curve reveals the signal’s complexity index (CI). A complex signal is associated with a time evolution with a rich structure on multiple scales. The first 10 s and the last 10 s – initiation and termination of 2-min treadmill walking (TW_1-3_) – were excluded from the analysis. The local average and the local SD of each time series were computed for each spatiotemporal parameter. Table 2 provides further details.

### 2.6. Statistical analyses

For all statistical comparisons, assumptions of ANOVA (e.g., homogeneity of variance and normal distribution) were tested using the normality and Leven’s tests. Correction for multiple follow-up comparisons was done using the Bonferroni correction. All other univariate analyses uses one-way split-plot repeated measures ANOVA with Greenhouse–Geisser correction for sphericity (i.e., between subject effect, training group, is the whole plot effect of a split-plot design). The Subject effect is nested within the Group effect which was specified as random.

The generalizability of two types of training programs (PST during stance and dynamic gait) and Pre-test and Post-test differences on dependent measures in Table 2 were ascertained using a linear mixed effect model on all gait and posture parameters using the above repeated measures ANOVA. The statical analyses were processed using the JMP Pro 16, 2021, SAS Institute.

## 3. Results

### 3.1. Effects of PST during stance on feedforward or proactive adaptation and, associated group effect (generalizability of two types of PST-stance and walking)

**Figure 5:**
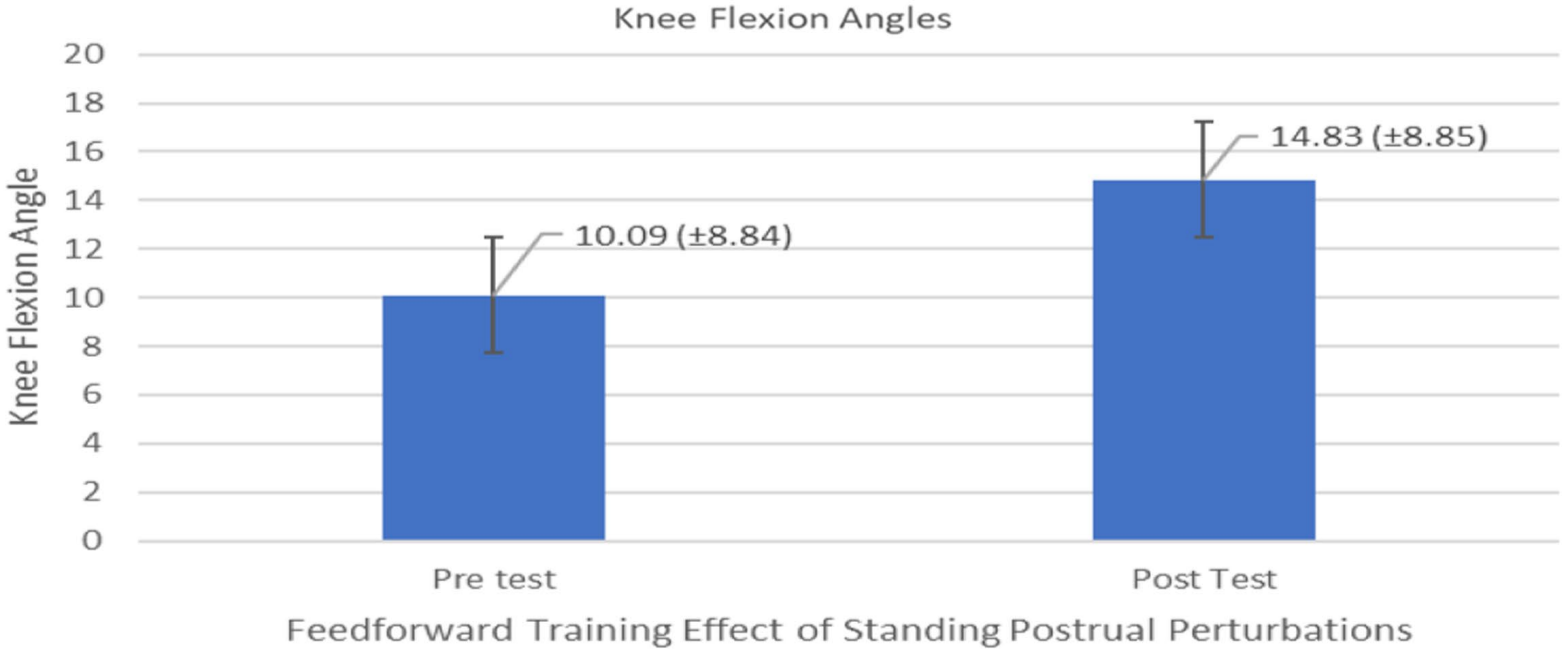
Feedforward Effects of PST during stance on the knee flexion angles with the means and standard deviations shown.

**Figure 6:**
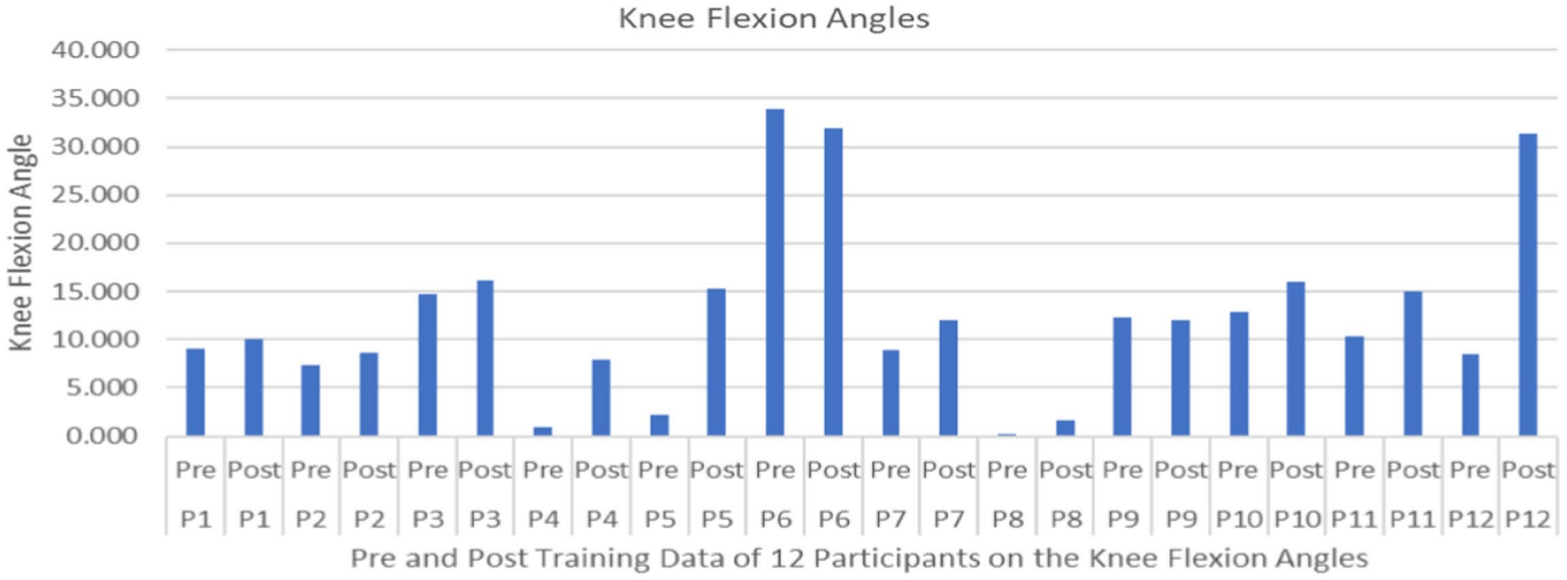
Feedforward Effects of PST during stance on the knee flexion angles of all participants.

The results of the univariate repeated measures ANOVAs on all dependent measures associated with feedforward variables in Table 2 indicated a significant differences on only one of the feedforward variables – Knee flexion angle during pre and post-trial period (*F*_1,10_ = 5.662, *p* = 0.0386, effect size 0.259) (Figure 5). No significant differences were observed for the group comparison or generalizability of two types of training (*F*_1,10_ = 0.0102, *p* = 0.92) on the knee flexion angle or all other feedforward dependent measures in Table 2. In general, knee flexion angles were significantly different indicating a proactive response using the knee strategy (i.e., bending the knee to lower the whole body COM) to maintain stability (Figure 6).

### 3.2. Effects of PST during stance on feedback or reactive adaptations

**Figure 7:**
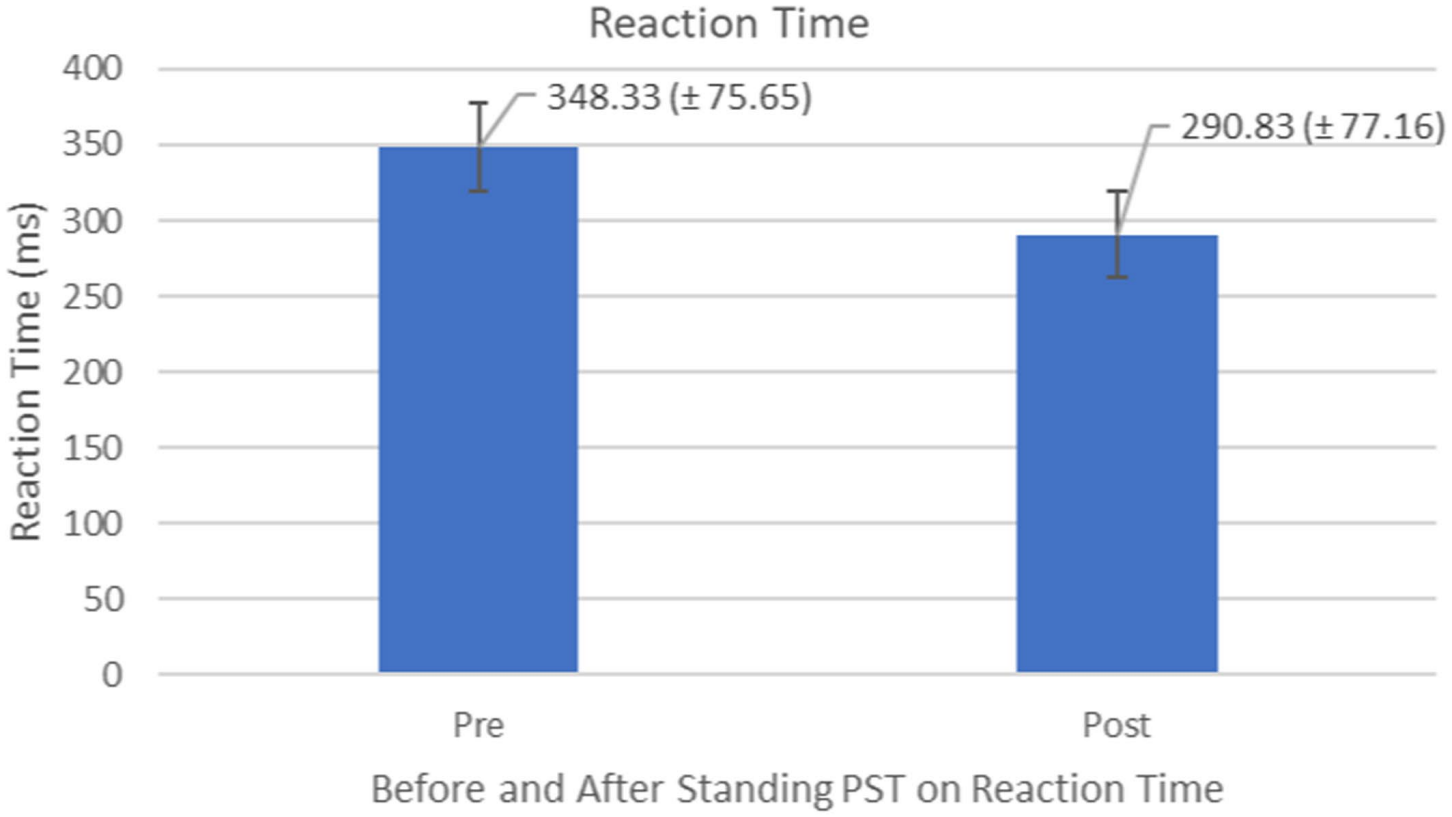
Feedback Effects of PST during stance on reaction time of the stance foot with the mean reaction times and standard deviations shown.

**Figure 8:**
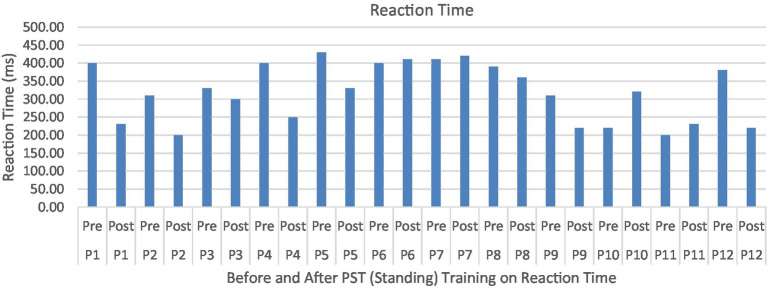
Reaction time or Time_Latency_ (ms) associated with postural perturbation training during the first and the last perturbations. Participants were able to produce quicker responses to postural perturbations after the PST.

Compared to the initial unexpected perturbation block, reaction time (Time_Latency_) showed significant improvement (*F*_1,10_ = 4.94, *p* = 0.050, effect size =0.352) (Figure 7). This suggests that reactive adaption utilizing feedforward mechanisms is still active and may be trained and directed toward improving fall safety (Figure 8). No significant group (generalizability of two types of training) effect (*F*_1,10_ = 0.012, *p* = 0.916) was observed for the reactive adaptation variables in Table 2.

### 3.3. Effects of PST during walking on feedforward or proactive adaptations and generalizability of training groups

**Figure 9:**
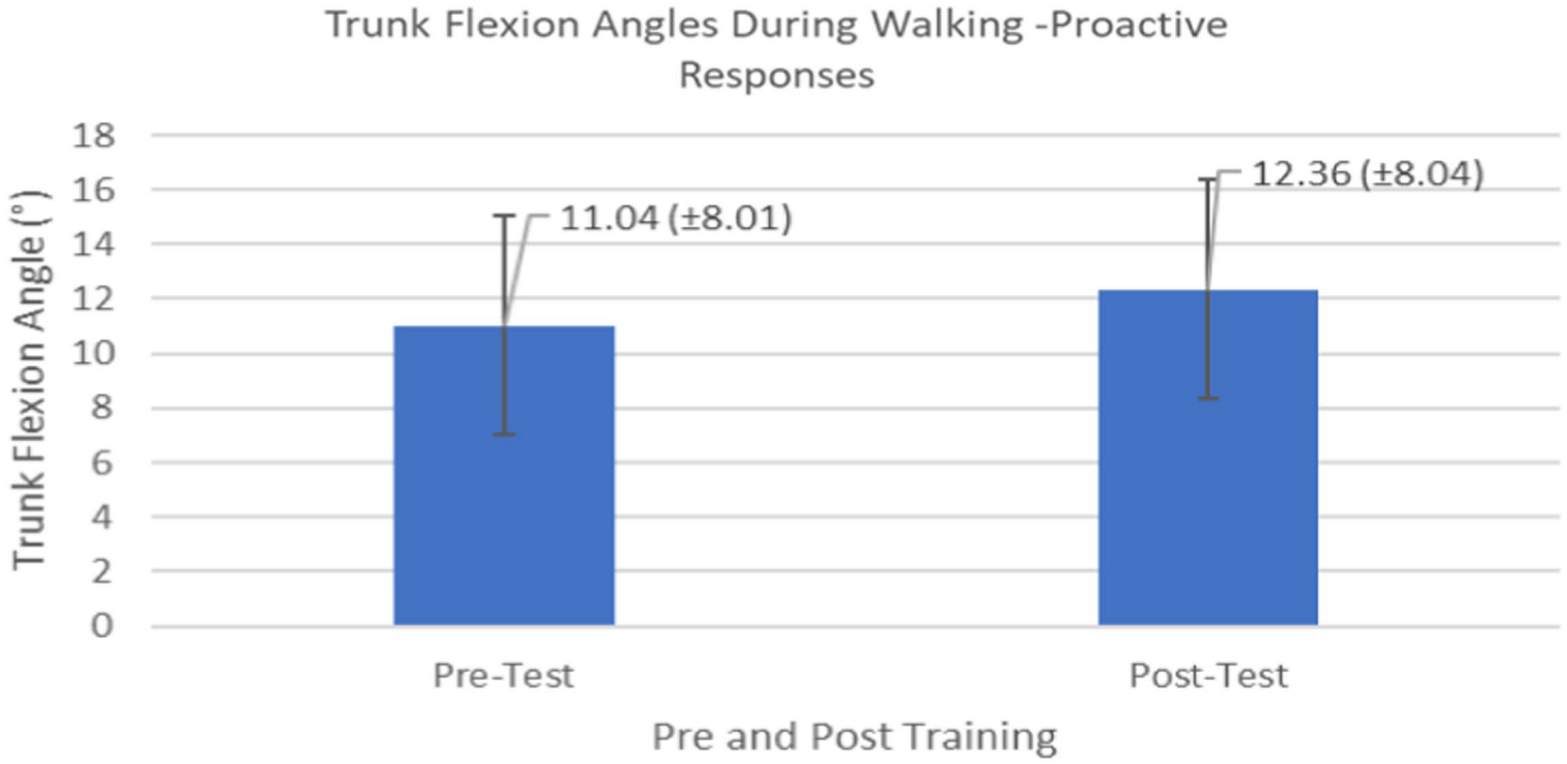
Feedforward Effects of gait perturbation training with the mean reaction times and standard deviations shown.

**Figure 10:**
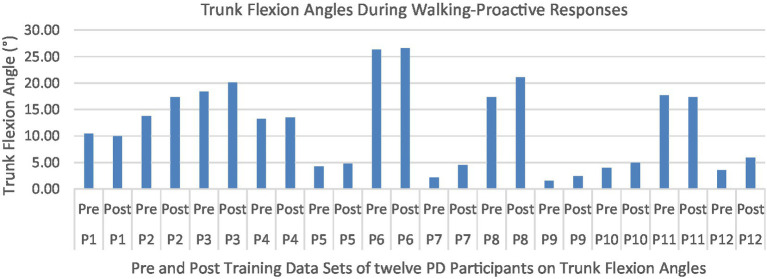
Individual effects of gait perturbation training on feedforward/proactive adaptation of trunk flexion.

**Figure 11:**
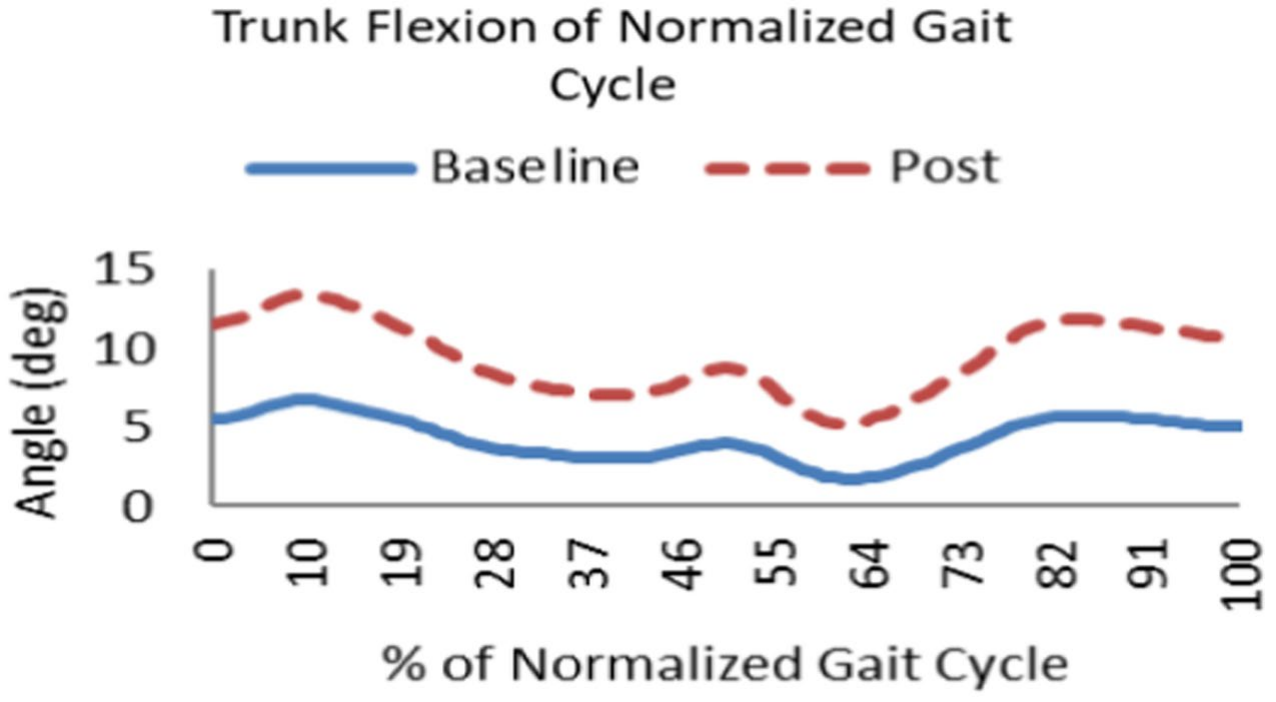
Following repeated gait perturbation training a subject’s trunk flexion is greater throughout the gait cycle exhibiting feedforward/proactive adaptation.

**Figure 12:**
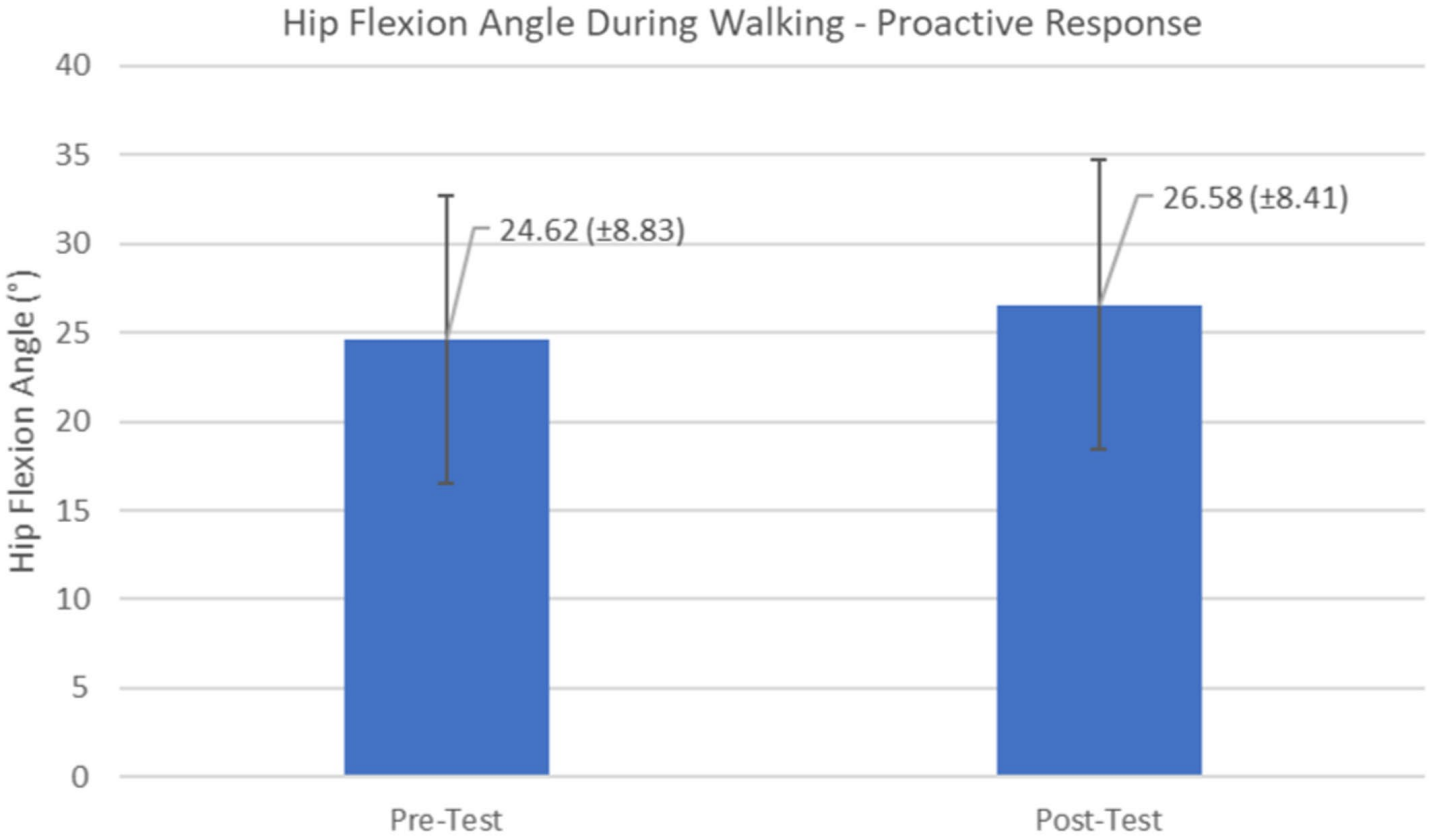
Feedforward Effects of gait perturbation training with the mean hip flexion angles and standard deviations shown.

**Figure 13:**
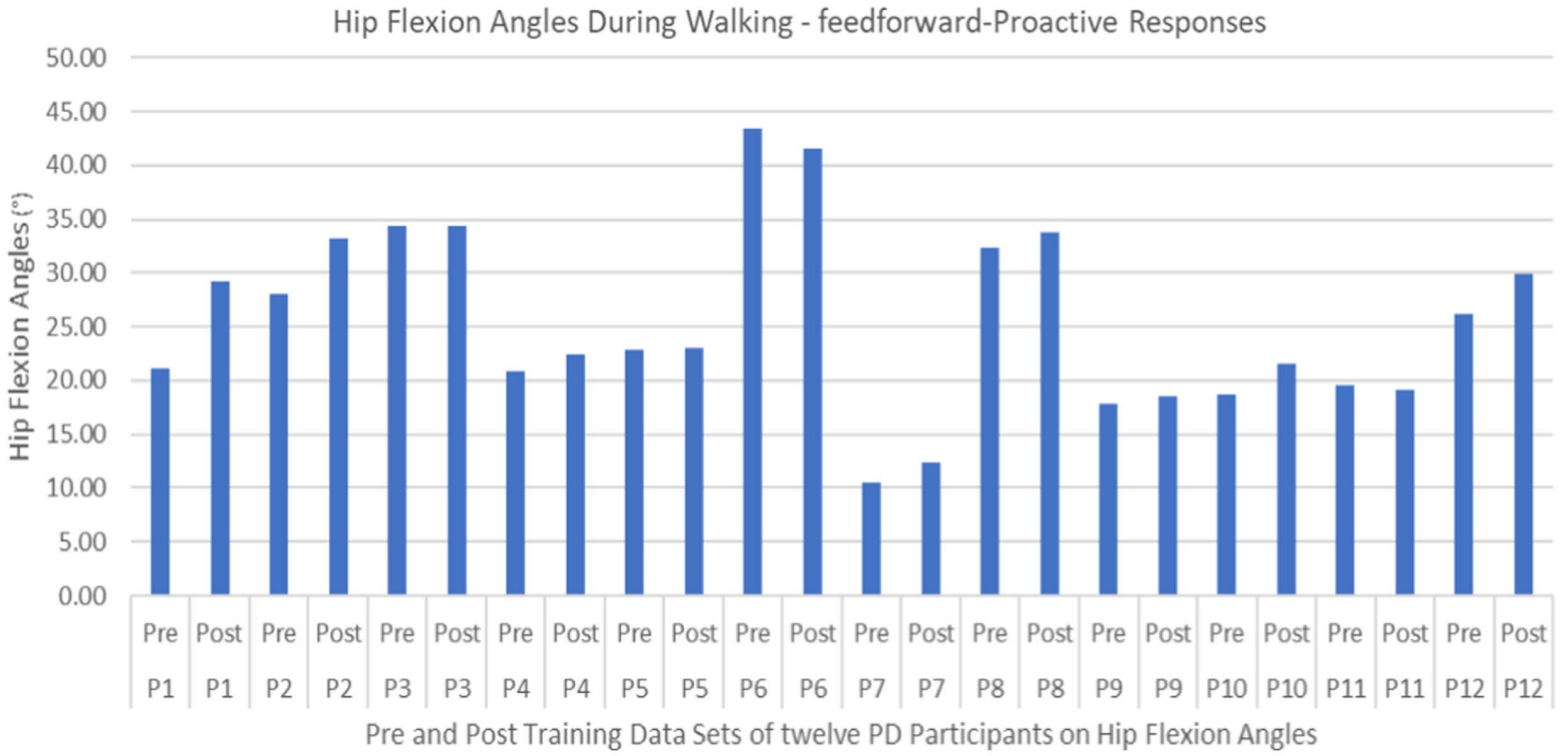
Individual effects of gait perturbation training on feedforward/proactive adaptation of hip flexion.

**Figure 14:**
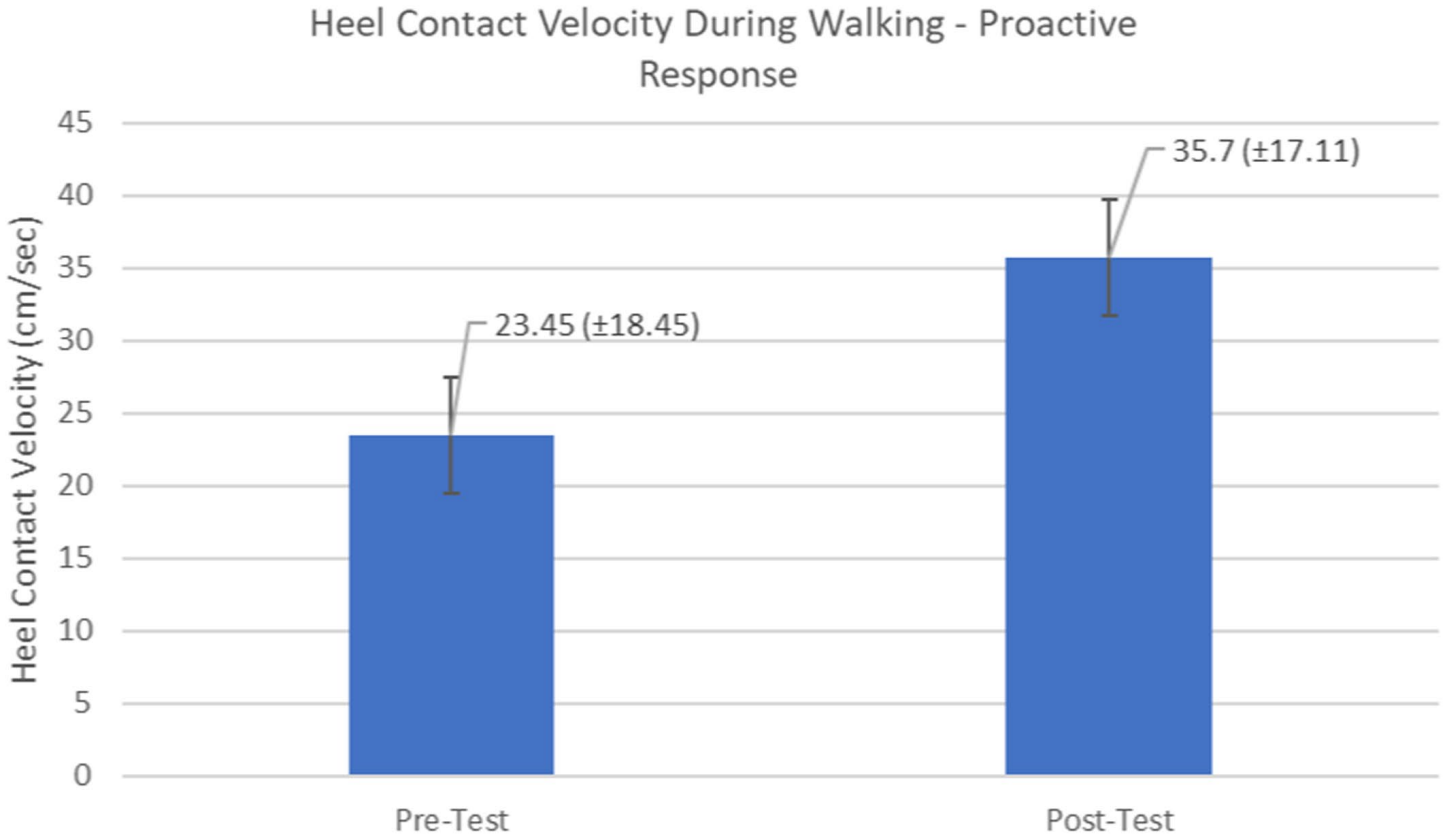
Feedforward Effects of gait perturbation training on heel contact velocity.

**Figure 15:**
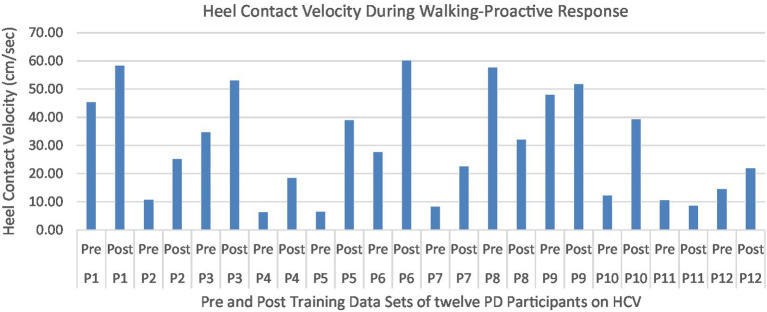
Individual effects of gait perturbation training on feedforward/proactive adaptation of heel contact velocity.

Proactive adaptations during gait were assessed during the 10–15 steps before the initial perturbation block (GP_1_) and the 10–15 steps preceding the last perturbation block (GP_12_). Flexion angles of the trunk, hip, and knee of the perturbed limb (pertb_hc_) were significantly greater from GP_1_ to GP_12_, revealing feedforward adaptations in anticipation of the perturbation. These biomechanical modifications are characterized by the varying trunk (*F*_1,10_ = 11.311, *p* = 0.007, effect size = 0.08) (Figures 9–11) and hip flexion angles (*F*_1,10_ = 5.709, *p* = 0.038, effect size = 0.05) (Figures 12, 13) and, adopting vigilant gait marked by higher heel contact velocity (*F*_1,10_ = 6.503, *p* = 0.029, effect size = 0.33) (Figures 14, 15). Group effects (generalizability of training) were not statistically significant for these variables (i.e., trunk and hip flexion angles and heel contact velocity).

### 3.4. Effects of PST during walking on feedback or reactive adaptations

No significant differences were observed in any of the reactive parameters associated with recovery from a slip.

## 4. Discussion

The primary goal of this study was to assess how PD affects the ability to respond to slip specific perturbations and if one session of protective step training can produce short-term adaptations to improve walking and static balance control. A secondary goal was to determine the generalizability of two types of training programs (PST during stance and dynamic gait) and to what extent the perturbation-based balance training are specific and transferable to the nature of the perturbations experienced. At present, little is known regarding the effect of PD on the ability to react and adapt to standing and walking perturbations. For example, previous studies have shown improved adaptive capacity with perturbation responses in the mediolateral direction (21, 32). However, the specific nature of the perturbations in the current study were to simulate realistic slip perturbations without any walking aid, which were performed by anterior translations during standing and walking. We hypothesized that participants with PD would experience difficulty adapting reactive feedback-based strategies, but would adapt to planned feedforward strategies to standing and dynamic walking perturbation programs. The results generally support this hypothesis, showing that participants with PD were able to use experiences with perturbation training to integrate and adapt proactive feedforward strategies. Reactive, feedback strategies were less frequently improved through practice in the current cohort. Notably, there was little generalization between in-place and walking practice. The ability of the participants with PD to adapt to changes in task demands, particularly proactive behavior, suggests that individuals with PD could benefit from a specific training paradigm to facilitate specific balance control during rehabilitation (13, 33).

The results in this study are consistent with previous studies regarding early PD, which reported proactive adjustments during postural stability and locomotor perturbation tasks (6, 7, 20). This was evident during the walking perturbation program when comparing the effects of walking behavior prior to the initial walking perturbation block (GP_1_) and the final perturbation block (GP_12_). Particpants demonstrated significant feedforward adaptations in anticipation of the unexpected perturbation by significantly increasing trunk and hip flexions during walking along with higher heel contact velocity to veer away from untimely balance perturbations and adopting a more cautious gait to increase stability. However, this proactive effort may not have been fully realized in the current study as we found no significant differences in any of the reactive measures for these patients.

Predictive control is associated with supraspinal structures (8) involving cognitive processes like attention and memory (9) that may not be impaired in the early stages of the disease (8, 9). Predictive responses are important components for safe locomotion (33, 34) because they reduce the consequences of expected perturbations (12) and ultimately reduce the risk of falls. Thus, the increased risk of falls in early PD patients may be associated with deficits in reactive motor control. Understanding the ability of someone with PD to adapt to changes in task-specific demands will be useful in therapeutic intervention strategies.

Studies have closely linked the striatal system to motor learning, (5, 35, 36), suggesting that individuals with deficiencies in this system, such as those with Parkinson’s disease, would, in addition to the degradation of their movement patterns at baseline, have difficulty acquiring movement schema that would allow them to learn tasks quickly and accurately. However, studies examining the ability of patients with PD to learn and adapt to motor tasks have been relatively inconsistent (10–13, 37–45). While these studies indicate that PD patients are able to learn motor tasks, there is disagreement about the amount and type of improvement. A possible reason for this is that conflicting studies utilized different types of learning (implicit and explicit), considering specific aspects of learning are more severely impacted by PD, especially in the early stages of the disease. It has been reported that PD patients are able to learn specific tasks, however, they may require more practice than healthy controls do. Furthermore, the learned skills are not easily generalizable to other tasks, even if those tasks are similar (5, 40–45). The slower rate of learning and lack of transference may imply that PD patients are still able to learn in an explicit, feedforward manner – they may “pre-program” specific techniques and tasks quite capably - i.e., proactive mechanisms – but they may be unable to easily adjust to changes requiring the use of automatic or reactive mechanisms, making ready adaptation to changing conditions or simultaneous completion of multiple tasks, both of which are often required for balance and gait, quite difficult.

### 4.1. Predictive motor adaptations of PST

Learning integration for both proactive (feedforward) and reactive (feedback) adaptations were analyzed from the gait and postural PST paradigm, Figure 1. Feedforward responses from gait were extracted from the 15 s of unperturbed walking prior to each perturbation. Trunk flexion and hip flexion of the perturbed limb (pertb_hc_) were significantly greater compared to baseline, revealing feedforward adaptations in anticipation of the perturbation. Furthermore, heel contact velocity was increased in an effort to regain balance given a perturbation. The gait modification demonstrates the adoption of a more considered and vigilant gait to increase stability.

### 4.2. Reactive motor adaptations of PST

However, even after a significant effort of the feedforward system, the reactive responses to gait PST were not robustly impacted through a single session of 12 perturbations. Regarding walking slips, recovery step time (Time_1stStep_) – the time elapsed between perturbation onset and the first recovery step of the contralateral foot – showed no significant difference between baseline and the last perturbation trial. Similarly, the recovery period (Time_Rec_) – time elapsed from perturbation onset until the zero-cross of the anteroposterior COM velocity – also did not show improvement in their reactive recovery time.

Like gait PST, reactive feedback control for static stance PST was not robustly improved through practice. This is in contrast with some previous results (46, 47). Alternatively, it is also possible that feedforward modifications may not have generated sufficient stability improvements post-perturbation adaptations to the stability margins. Finally, it is possible that 12 perturbations was too little of a dose to produce meaningful improvements as were observed in previous studies.

### 4.3. Perturbation-specific transference

The secondary goal of this study was to determine the generalizability of PST during stance and PST during dynamic gait, and to what extent the specific type of the perturbations may transfer to perturbations in everyday situations. Dopaminergic treatments has been shown to be ineffective or unsatisfactory at treating postural instability and gait dysfunction in idiopathic PD, however, studies have demonstrated that therapy explicitly focusing on posture, gait, and balance may significantly improve these factors (18, 32). It is hypothesized that therapy specifically modeling situations in which individuals with PD are likely to fall (e.g., slipping due to shuffled steps/reduced executive function) may be more beneficial to prevent future falls than more generalized physical therapy. Further, repeated perturbation training has been shown in previous studies to improve features of postural stability and gait in PD patients. However, most of this preceding work has focused primarily on ascertaining the effects of training during static stance, either through training of center of pressure shifts toward a patient’s limits of stability (teaching the patient to weight-shift and lean safely) (48, 49) or through the training of adaptive responses (either postural adjustments or compensatory stepping) to regain balance following an external perturbation (32, 46, 50–52). These studies suggest that PD patients are able to learn to adapt to perturbations, that these adaptations may persist for several months, and that PST may enhance balance confidence. However, the generalizability of response improvements to other types of perturbation, such as during standing and walking, is uncertain (40–45). Results from the current study suggested that there may be limited generalizability across two types of training programs (PST during stance and dynamic gait). Albeit, healthy older adults were able to genderized their training (slips/trips) (53), in this study with PD patients, the specific training (either postural or gait perturbation training) did not improve balance in the untrained task. Thus, more personalized and specific training program is required to improve balance maintenance in PD patients.

## 5. Limitations

Several limitation to this study should be considered when interpreting the findings. First, this was a preliminary study to elucidate the optimal dose and frequency, as well as the therapeutic index, to determine maximal efficacy for a PST dose–response relationship in PD populations. The present study applies only a single perturbation acceleration of 10 m/s^2^ in the anterior direction to elicit a slip-specific perturbation effect. We also did not use a younger or age-matched control group to be compared. This perturbation was chosen to represent a worst-case scenario condition for PD and as an incipient marker for a perturbation scenario and population that is not well researched in the literature. Given the preliminary nature of this study, our sample size was relatively small (post effect sizes were from 0.03 to 0.352) and may affect the strength of our conclusions. Given the heterogeneity of PD, further studies involving a larger number and a wider range of PD participants is warranted. Further, the present finding only looked at acute after-effects of compensatory and adaptive behavior modifications, directly after the intervention. The authors did not perform repeated measurements over a period of time to determine the efficacy of the adaptive responses and do not expect the after-effects to imprint over a longitudinal period. Further, observed feedback and feedforward behavior may be dependent on the specific type of the perturbations experienced and may not show transfer to other forms of perturbations. Future studies will investigate the longevity of the acute after-effects produced in the present study. Finally, these effects were associated with PD patients’ average H&Y score of 2.7, and further study linking these two assessments to create a personalized treatment program is highly recommended.

## 6. Conclusion

PST is an efficient and effective way to discern reactive and proactive responses to therapeutic intervention. It has been suggested that this task-specific training approach may present a paradigm shift in fall prevention. While PD patients are still able to improve performance with practice, particularly in feedforward aspects of postural responses, reactive aspects of postural responses were not uniformly improved through practice. Because of this, patients with PD may require more training to achieve and retain motor learning and may require additional sensory information or motor guidance in order to facilitate this learning. These shortcomings in motor learning in PD could contribute to the degeneration in gait and balance often seen in the disease, as patients are unable to adapt to the gradual sensory and motor degradation. Research has shown that physical and exercise therapy can help PD patients to adapt new feedforward strategies to partially counteract these symptoms. In particular, balance, treadmill, resistance, and repeated perturbation training (PST) therapies have been shown to improve motor patterns in PD. However, much research is still needed to determine which of these therapies best alleviates which symptoms of PIGD, the needed dose and intensity of these therapies, the long-term retention effects, and the benefits of such technologies as augmented feedback, motorized perturbations, virtual reality, and weight-bearing assistance.

## Data availability statement

The raw data supporting the conclusions of this article will be made available by the authors, without undue reservation.

## Ethics statement

The studies involving humans were approved by Arizona State University Institutional Review Board. The studies were conducted in accordance with the local legislation and institutional requirements. The participants provided their written informed consent to participate in this study.

## Author contributions

TL was responsible for compiling the sources used within this study and writing the drafted manuscript. TL, MO, and AL provided additional articles, discussed possible implications, and revised the manuscript. All authors contributed to the article and approved the submitted version.
